# Supplementary material for: Backscattering-free edge states below all bands in two-dimensional auxetic media
Source: Nat Commun. 2025 Mar 10;16:2373. doi: 10.1038/s41467-025-57518-x (PMC11893909; doi:10.1038/s41467-025-57518-x)
Supplement: Supplementary file 1 — Supplementary Information [file 41467_2025_57518_MOESM1_ESM.pdf]

Backscattering-free edge states below all bands in two-dimensional auxetic media: Supplementary Materials

The supplementary materials contain detailed mathematical discussion on 2D and 3D Rayleigh wave modes, one-way transport criteria for continuum and discrete systems, the spin and momentum of elastic waves, as well as the full spin and momentum locking of Rayleigh waves in 2D materials with $B/\mu \ll 1$.

SI. 3D RAYLEIGH WAVES

Here we review the linear elastic wave equation and Rayleigh wave formula in 3D. The main references for this formulation are Ref. [1, 2]. Consider a homogeneous elastic material in the $x^3 \leq 0$ region of a x^1-x^2-x^3 space with Lamé constants μ and $\lambda_{3\mathrm{D}} = B_{3\mathrm{D}} - \frac{2}{3}\mu$, where $B_{3\mathrm{D}}$ is the bulk modulus. We use the convention herein where the subscript 3D denotes the properties of 3D media, and no subscript denotes the effective property of 2D media or quantities where they are the same for both. Let $\mathbf{v}$ be the displacement vector field. The strain tensor is given by

$$u_{jk} = \frac{1}{2}\left(\delta_{ik}\frac{\partial v^i}{\partial x^j} + \delta_{ij}\frac{\partial v^i}{\partial x^k}\right). \tag{S1}$$

The elastic energy density for homogeneous material in 3D is

$$\epsilon_{3\mathrm{D}} = \frac{1}{2}\lambda_{3\mathrm{D}}\delta^{ij}\delta^{kl}u_{ij}u_{kl} + \mu\delta^{ij}\delta^{kl}u_{ik}u_{jl} \tag{S2}$$

The stress tensor σ^{ij} is defined as

$$d\epsilon = \sigma^{ij}du_{ij}, \tag{S3}$$

Applying $\epsilon = \epsilon_{3\mathrm{D}}$ in (S3), we obtain the stress tensor for 3D media

$$\sigma^{ij}_{3\mathrm{D}} = \lambda_{3\mathrm{D}}\delta^{ij}\frac{\partial v^a}{\partial x^a} + \mu\left(\delta^{ib}\frac{\partial v^j}{\partial x^b} + \delta^{jc}\frac{\partial v^i}{\partial x^c}\right). \tag{S4}$$

Let ρ be the mass density of the elastic material. The equation of motion in 3D is obtained via Newton's second law

$$\rho\frac{\partial^2 v^i}{\partial t^2} = \frac{\partial \sigma^{ij}_{3\mathrm{D}}}{\partial x^j}, \tag{S5}$$

which is equivalent to

$$\rho\frac{\partial^2 \mathbf{v}}{\partial t^2} = (\lambda_{3\mathrm{D}} + \mu)\,\boldsymbol{\nabla}\boldsymbol{\nabla}\cdot\mathbf{v} + \mu\nabla^2\mathbf{v}. \tag{S6}$$

Using the Helmholtz decomposition

$$\mathbf{v} = \mathbf{v}_\mathrm{l} + \mathbf{v}_\mathrm{t}, \tag{S7}$$

where $\mathbf{v}_\mathrm{l}$ is the curl free and $\mathbf{v}_\mathrm{t}$ the divergence free parts, and taking curl on both sides of (S6), we get

$$\boldsymbol{\nabla}\times\left(\rho\frac{\partial^2 \mathbf{v}_\mathrm{t}}{\partial t^2} - \mu\nabla^2\mathbf{v}_\mathrm{t}\right) = 0, \tag{S8}$$

resulting in the wave equation for transverse waves

$$\frac{\partial^2 \mathbf{v}_\mathrm{t}}{\partial t^2} - c_\mathrm{t}^2\nabla^2\mathbf{v}_\mathrm{t} = 0, \tag{S9}$$

and, similarly, by taking gradient of (S6), we obtain the equation for 3D longitudinal waves

$$\frac{\partial^2 \mathbf{v}_\mathrm{l}}{\partial t^2} - c_{\mathrm{l},3\mathrm{D}}^2\nabla^2\mathbf{v}_\mathrm{l} = 0, \tag{S10}$$

where $c_{\mathrm{l,3D}} = \sqrt{\frac{\lambda_{\mathrm{3D}}+2\mu}{\rho}}$ and $c_{\mathrm{t}} = \sqrt{\frac{\mu}{\rho}}$ are the speed of longitudinal wave and transverse waves, respectively.

Assuming a surface wave propagating in the x^1 direction, we write the longitudinal and transverse displacement components of the Rayleigh wave $\mathbf{v}_{\mathrm{l}}$ and $\mathbf{v}_{\mathrm{t}}$, respectively, as

$$\begin{aligned}\mathbf{v}_{\mathrm{l,3D}} &= e^{i\left(k_{\mathrm{3D}}x^1-\omega t\right)+\kappa_{\mathrm{l,3D}}x^3}\left(\alpha^1_{\mathrm{3D}}, \alpha^2_{\mathrm{3D}}, \alpha^3_{\mathrm{3D}}\right)^T,\\ \mathbf{v}_{\mathrm{t,3D}} &= e^{i\left(k_{\mathrm{3D}}x^1-\omega t\right)+\kappa_{\mathrm{t,3D}}x^3}\left(\beta^1_{\mathrm{3D}}, \beta^2_{\mathrm{3D}}, \beta^3_{\mathrm{3D}}\right)^T.\end{aligned} \tag{S11}$$

Plugging (S11) into (S9), (S10), we obtain

$$\kappa_{\mathrm{l,3D}} = \sqrt{k^2_{\mathrm{3D}} - \frac{\omega^2}{c^2_{\mathrm{l,3D}}}},\ \kappa_{\mathrm{t,3D}} = \sqrt{k^2_{\mathrm{3D}} - \frac{\omega^2}{c^2_{\mathrm{t}}}}, \tag{S12}$$

Given the boundary conditions at the free surface

$$\begin{aligned}\sigma^{13}_{\mathrm{3D}} &= \mu\left(\frac{\partial v^3}{\partial x^1} + \frac{\partial v^1}{\partial x^3}\right) = 0,\\ \sigma^{23}_{\mathrm{3D}} &= \mu\left(\frac{\partial v^3}{\partial x^2} + \frac{\partial v^2}{\partial x^3}\right) = 0,\\ \sigma^{33}_{\mathrm{3D}} &= \lambda_{\mathrm{3D}}\frac{\partial v^1}{\partial x^1} + \lambda_{\mathrm{3D}}\frac{\partial v^2}{\partial x^2} + \left(\lambda_{\mathrm{3D}} + 2\mu\right)\frac{\partial v^3}{\partial x^3} = 0\end{aligned} \tag{S13}$$

and the conditions $\nabla \times \mathbf{v}_{\mathrm{l}} = 0$ and $\nabla \cdot \mathbf{v}_{\mathrm{t}} = 0$ (independent of 2D vs. 3D), we obtain

$$\begin{aligned}-\kappa_{\mathrm{l,3D}}\alpha^1_{\mathrm{3D}} + ik_{\mathrm{3D}}\alpha^3_{\mathrm{3D}} &= 0,\\ ik_{\mathrm{3D}}\beta^1_{\mathrm{3D}} + \kappa_{\mathrm{t,3D}}\beta^3_{\mathrm{3D}} &= 0\\ \alpha^2_{\mathrm{3D}} = \beta^2_{\mathrm{3D}} &= 0\end{aligned} \tag{S14}$$

which says there exists a_{3D} and B_{3D} such that

$$\begin{aligned}\alpha^1_{\mathrm{3D}} &= k_{\mathrm{3D}}a_{\mathrm{3D}},\ \alpha^3_{\mathrm{3D}} = -i\kappa_{\mathrm{l,3D}}a_{\mathrm{3D}},\\ \beta^1_{\mathrm{3D}} &= \kappa_{\mathrm{t,3D}}B_{\mathrm{3D}},\ \beta^3_{\mathrm{3D}} = -ik_{\mathrm{3D}}B_{\mathrm{3D}}.\end{aligned} \tag{S15}$$

Combining (S11), (S13) and (S15) we get

$$\begin{aligned}B_{\mathrm{3D}}\left(k^2_{\mathrm{3D}} + \kappa^2_{\mathrm{t,3D}}\right) + 2a_{\mathrm{3D}}k_{\mathrm{3D}}\kappa_{\mathrm{l,3D}} &= 0,\\ 2B_{\mathrm{3D}}\kappa_{\mathrm{t,3D}}k_{\mathrm{3D}} + a_{\mathrm{3D}}\left(k^2_{\mathrm{3D}} + \kappa^2_{\mathrm{t,3D}}\right) &= 0.\end{aligned} \tag{S16}$$

In order that (S16) has a non-zero solution, the coefficient matrix need to be of determinant zero, which gives

$$\left(k^2_{\mathrm{3D}} + \kappa^2_{\mathrm{t,3D}}\right)^2 = 4k^2_{\mathrm{3D}}\kappa_{\mathrm{t,3D}}\kappa_{\mathrm{l,3D}}. \tag{S17}$$

Expressing (S17) in terms of wavespeeds, with the 3D Rayleigh wavespeed $c_{\mathrm{3D}} = \omega/k$, we obtain the well known Rayleigh equation

$$\left(2 - c^2_{\mathrm{3D}}/c^2_{\mathrm{t}}\right)^2 = 4\left(1 - c^2_{\mathrm{3D}}/c^2_{\mathrm{l}}\right)^{1/2}\left(1 - c^2_{\mathrm{3D}}/c^2_{\mathrm{t}}\right)^{1/2}. \tag{S18}$$

Letting $\xi_{\mathrm{3D}} = c_{\mathrm{3D}}/c_{\mathrm{t}}$, we can further express (S18) as

$$\xi^6_{\mathrm{3D}} - 8\xi^4_{\mathrm{3D}} + 8\xi^2_{\mathrm{3D}}\left(3 - \frac{6}{3h_{\mathrm{3D}} + 4}\right) - 16\left(1 - \frac{3}{3h_{\mathrm{3D}} + 4}\right) = 0, \tag{S19}$$

where $h_{\mathrm{3D}} = \frac{B_{\mathrm{3D}}}{\mu}$. This can also be rewritten as

$$\xi^6_{\mathrm{3D}} - 8\xi^4_{\mathrm{3D}} + \xi^2_{\mathrm{3D}}\left(24 - 16\frac{c^2_{\mathrm{t}}}{c^2_{\mathrm{l,3D}}}\right) - 16\left(1 - \frac{c^2_{\mathrm{t}}}{c^2_{\mathrm{l,3D}}}\right) = 0. \tag{S20}$$

The corresponding modes at the $x^3 = 0$ surface are

$$\begin{aligned}
\boldsymbol{\psi}_{\mathbf{k},3\mathrm{D}}(x^1,x^2,0) &= e^{i(k_{3\mathrm{D}}x^1-\omega t)}\left(-\frac{\xi_{3\mathrm{D}}^2\sqrt{1-\xi_{3\mathrm{D}}^2}}{2-\xi_{3\mathrm{D}}^2},0,i\left(\frac{2\sqrt{1-\xi_{3\mathrm{D}}^2}}{2-\xi_{3\mathrm{D}}^2}\sqrt{1-\frac{3\xi_{3\mathrm{D}}^2}{3h_{3\mathrm{D}}+4}}-1\right)\right)^T, \\
\boldsymbol{\psi}_{-\mathbf{k},3\mathrm{D}}(x^1,x^2,0) &= e^{i(-k_{3\mathrm{D}}x^1-\omega t)}\left(-\frac{\xi_{3\mathrm{D}}^2\sqrt{1-\xi_{3\mathrm{D}}^2}}{2-\xi_{3\mathrm{D}}^2},0,-i\left(\frac{2\sqrt{1-\xi_{3\mathrm{D}}^2}}{2-\xi_{3\mathrm{D}}^2}\sqrt{1-\frac{3\xi_{3\mathrm{D}}^2}{3h_{3\mathrm{D}}+4}}-1\right)\right)^T.
\end{aligned} \tag{S21}$$

SII. 2D RAYLEIGH WAVES

Here we review the elastic wave equation and Rayleigh wave formula for a flat thin homogeneous elastic sheet within a plane stress condition, with bulk modulus $B_{3\mathrm{D}}$ and Lamé constants μ and $\lambda_{3\mathrm{D}}$. Equivalent results were previously derived in Ref. [3]. We choose a coordinate system such that the x^1 and x^2 directions lie in the plane of the sheet and the x^3 direction is normal to the plane. On the top and bottom surfaces of the sheet, we have

$$\sigma^{13} = \sigma^{23} = \sigma^{33} = 0, \tag{S22}$$

constituting a plane stress state. By the continuity of stress tensor and assuming the thickness is small, we can assume $\sigma^{13} = \sigma^{23} = \sigma^{33} = 0$ holds everywhere. Making use of (S22) and (S2), the energy density in this situation is

$$\epsilon = \frac{1}{2}\lambda\delta^{ij}\delta^{kl}u_{ij}u_{kl} + \mu\delta^{ij}\delta^{kl}u_{ik}u_{jl}, \tag{S23}$$

where i, j ranges from 1 to 2 and the effective 2D 1st Lamé constant is

$$\lambda = \frac{2\left(B_{3\mathrm{D}} - \frac{2}{3}\mu\right)\mu}{B_{3\mathrm{D}} + \frac{4}{3}\mu}. \tag{S24}$$

The effective 2D bulk modulus is thus

$$B = \lambda + \mu = \frac{3B_{3\mathrm{D}}\mu}{B_{3\mathrm{D}} + \frac{4}{3}\mu}. \tag{S25}$$

We note here that if $B_{3\mathrm{D}} = 0$ then $B = 0$ also. Letting $\mathbf{v} = v^1x^1 + v^2x^2$ be the in-plane displacement, and noting (S23) has the same form as (S2), the equation of motion for $\mathbf{v}$ is then

$$\rho\frac{\partial^2\mathbf{v}}{\partial t^2} = (\lambda+\mu)\boldsymbol{\nabla}\boldsymbol{\nabla}\cdot\mathbf{v} + \mu\nabla^2\mathbf{v}. \tag{S26}$$

We simplify the equation again using the Helmholtz decomposition, giving

$$\begin{aligned}
\rho\frac{\partial^2\mathbf{v}_\mathrm{t}}{\partial t^2} - \mu\nabla^2\mathbf{v}_\mathrm{t} &= 0, \\
\rho\frac{\partial^2\mathbf{v}_\mathrm{l}}{\partial t^2} - (\lambda+2\mu)\nabla^2\mathbf{v}_\mathrm{l} &= 0.
\end{aligned} \tag{S27}$$

Now we calculate Rayleigh waves in the aforementioned thin sheet, but modified into a half-sheet laying in $x^2 \leq 0$. We write the longitudinal and transverse components of the Rayleigh wave as

$$\begin{aligned}
\mathbf{v}_\mathrm{l} &= e^{i(kx^1-\omega t)+\kappa_\mathrm{l}x^2}\left(\alpha^1,\alpha^2\right)^T, \\
\mathbf{v}_\mathrm{t} &= e^{i(kx^1-\omega t)+\kappa_\mathrm{t}x^2}\left(\beta^1,\beta^2\right)^T.
\end{aligned} \tag{S28}$$

Plugging (S28) into (S27), we obtain

$$\kappa_{\mathrm{l}} = \sqrt{k^2 - \frac{\omega^2}{c_{\mathrm{l}}^2}},\ \kappa_{\mathrm{t}} = \sqrt{k^2 - \frac{\omega^2}{c_{\mathrm{t}}^2}}, \tag{S29}$$

where $c_{\mathrm{l}} = \sqrt{\frac{\lambda+2\mu}{\rho}}$ is the effective longitudinal wavespeed in the plate. The $x^2 = 0$ line being an open boundary gives us the boundary conditions

$$\begin{aligned} \sigma^{12} &= \mu\left(\frac{\partial v^1}{\partial x^2} + \frac{\partial v^2}{\partial x^1}\right) = 0, \\ \sigma^{22} &= \lambda\frac{\partial v^1}{\partial x^1} + (\lambda + 2\mu)\frac{\partial v^2}{\partial x^2} = 0. \end{aligned} \tag{S30}$$

Plugging (S28) into (S30), we get

$$\begin{aligned} &\kappa_{\mathrm{l}}\alpha^1 + ik\alpha^2 + \kappa_{\mathrm{t}}\beta^1 + ik\beta^2 = 0, \\ &\frac{ik\lambda}{\lambda + 2\mu}\alpha^1 + \kappa_{\mathrm{l}}\alpha^2 + \frac{ik\lambda}{\lambda + 2\mu}\beta^1 + \kappa_{\mathrm{t}}\beta^2 = 0. \end{aligned} \tag{S31}$$

The curl free condition of $\mathbf{v}_{\mathrm{l}}$ and divergence free condition of $\mathbf{v}_{\mathrm{t}}$ gives us

$$\begin{aligned} -\kappa_{\mathrm{l}}\alpha^1 + ik\alpha^2 &= 0, \\ ik\beta^1 + \kappa_{\mathrm{t}}\beta^2 &= 0. \end{aligned} \tag{S32}$$

Considering Eqs. S31 and S32, we have 4 linear equations for 4 variables $\alpha^1, \alpha^2, \beta^1, \beta^2$. In order that the equations have non-zero solutions, the corresponding coefficient matrix needs to be of determinant zero, which gives

$$\xi^6 - 8\xi^4 + \left(24 - 16\frac{c_{\mathrm{t}}^2}{c_{\mathrm{l}}^2}\right)\xi^2 - 16\left(1 - \frac{c_{\mathrm{t}}^2}{c_{\mathrm{l}}^2}\right) = 0, \tag{S33}$$

where $\xi = c/c_{\mathrm{t}}$, with c being the Rayleigh wave speed in the 2D plate. We note that Rayleigh wavespeeds c and $c_{3\mathrm{D}}$ are denoted as c_{R} in the main text. We see that (S33) is the same as (S20), with the exception that c_{l} replaces $c_{\mathrm{l,3D}}$, as was pointed out in Ref. [3]. In terms of $h = B/\mu$, ξ satisfies

$$\xi^6 - 8\xi^4 + \left(8 + \frac{16h}{1+h}\right)\xi^2 - \frac{16h}{1+h} = 0. \tag{S34}$$

The corresponding modes at the $x^2 = 0$ line are

$$\begin{aligned} \boldsymbol{\psi}_{\mathbf{k}}(x^1, 0) &= e^{i\left(kx^1 - \omega t\right)}\left(\frac{1}{2}\xi\sqrt{1 + \frac{1}{h}}, \frac{1}{4}i\xi\left(2 - \xi^2\right)\sqrt{\frac{1+h}{h - h\xi^2}}\right)^T, \\ \boldsymbol{\psi}_{-\mathbf{k}}(x^1, 0) &= e^{i\left(-kx^1 - \omega t\right)}\left(\frac{1}{2}\xi\sqrt{1 + \frac{1}{h}}, -\frac{1}{4}i\xi\left(2 - \xi^2\right)\sqrt{\frac{1+h}{h - h\xi^2}}\right)^T. \end{aligned} \tag{S35}$$

The decay factor κ_{t} can be rewritten in terms of ξ as:

$$\kappa_{\mathrm{t}} = k^2\sqrt{1 - \xi^2}, \tag{S36}$$

where a decrease in B/μ leads to a decrease in ξ (as seen in Fig. 1(g) in the main text), resulting in an increase in κ_{t}. Consequently, the decay length becomes shorter, and the Rayleigh wave becomes more localized.

The solution of (S34) yields three roots. Selecting the smallest positive root of ξ^2 and performing a series expansion about $h = 0$ ($B/\mu \to 0$), for up to order h we obtain $\xi \approx \sqrt{2h}$. Because, $c = \xi c_{\mathrm{t}}$, we can then approximate the Rayleigh wave speed as $c \approx \sqrt{2B/\rho}$ as $\nu \to -1$ (equivalently $B/\mu \to 0$), as seen in Fig. 1(j).

SIII. LINEAR RESPONSE TO POINT SOURCE ON THE EDGE

Here we derive the linear response theory for both 2D continuum and discrete systems (the same derivation also applies to 3D systems) for systems subject to a point source of vibration on the edge.

A. Continuum version

Considering the same homogeneous elastic thin sheet as described in Sec SII, we apply a local driving force $\mathbf{F}(\mathbf{r}, t) = Re\left(\mathbf{F}e^{-i\omega t}\right)\delta(\mathbf{r})$ to the boundary of the system, specifically at the $(0, 0)$ point. The complex equation of motion is then

$$\begin{aligned} \rho\frac{\partial^2\mathbf{v}}{\partial t^2} &= (\lambda+\mu)\nabla\nabla\cdot\mathbf{v} + \mu\nabla^2\mathbf{v} + \mathbf{F}\delta(\mathbf{r})e^{-i\omega t}, \\ \mathbf{v}(\mathbf{r}, 0) &= \frac{\partial\mathbf{v}(\mathbf{r}, 0)}{\partial t} = 0. \end{aligned} \tag{S37}$$

Let $\boldsymbol{\psi}_{\mathbf{k}}(\mathbf{r})e^{-i\omega_{\mathbf{k}}t}$ be 2D Rayleigh waves with wave vector $\mathbf{k}$ and $\boldsymbol{\phi}_s(\mathbf{r}, t)$ be bulk waves with index s (since the bulk waves are not important here we use an arbitrary index to identify them). The solution of (S37) can then be decomposed into

$$\mathbf{v}(\mathbf{r}, t) = \int_{\mathbf{k}} d\mathbf{k}C_{\mathbf{k}}(t)\boldsymbol{\psi}_{\mathbf{k}}(\mathbf{r})e^{-i\omega_{\mathbf{k}}t} + \int_s dsG_s(t)\boldsymbol{\phi}_s(\mathbf{r}, t). \tag{S38}$$

Plugging (S38) into (S37), applying $\boldsymbol{\psi}_{\mathbf{k}}(\mathbf{r})e^{-i\omega_{\mathbf{k}}t}$ to both sides, and doing an integration over time and position, we obtain

$$\begin{aligned} \frac{d^2C_{\mathbf{k}}(t)}{dt^2} - 2i\omega_{\mathbf{k}}\frac{dC_{\mathbf{k}}(t)}{dt} &= \boldsymbol{\psi}_{\mathbf{k}}(0)^*\cdot\mathbf{F}\; e^{-i\left(\omega-\omega_j(\mathbf{k})\right)t}, \\ \left.\frac{dC_{\mathbf{k}}(t)}{dt}\right|_{t=0} &= C_{\mathbf{k}}(0) = 0. \end{aligned} \tag{S39}$$

For a given k, if $\omega \neq \omega_{\mathbf{k}}$, $C_{\mathbf{k}}(t)$ is bounded. However, if $\omega = \omega_{\mathbf{k}}$, we have

$$C_{\mathbf{k}}(t) = \frac{i\boldsymbol{\psi}^*_{\mathbf{k}}(0)\cdot\mathbf{F}}{\omega}t + q_{\mathbf{k}}(t), \tag{S40}$$

where $q_{\mathbf{k}}(t)$ is bounded. Therefore, after long times, most energy is in the two modes $\boldsymbol{\psi}_{\mathbf{k}}(\mathbf{r})e^{-i\omega_{\mathbf{k}}t}$ and $\boldsymbol{\psi}_{-\mathbf{k}}(\mathbf{r})e^{-i\omega_{\mathbf{k}}t}$. To maximally excite the $+\mathbf{k}$ Rayleigh wave, the force applied to system needs to follow the trajectory of the motion corresponding to $\boldsymbol{\psi}_{\mathbf{k}}(\mathbf{r})e^{-i\omega_{\mathbf{k}}t}$, which means $\boldsymbol{\psi}_{\mathbf{k}}(0) = \mathbf{F}$. In this case, the ratio $|C_{-\mathbf{k}}/C_{\mathbf{k}}|$, whose square quantifies the ratio between energy going in $+\mathbf{k}$ direction and $-\mathbf{k}$ direction, is then

$$\left|\frac{C_{-\mathbf{k}}}{C_{\mathbf{k}}}\right| = \left|\frac{\boldsymbol{\psi}_{-\mathbf{k}}(0)^*\cdot\boldsymbol{\psi}_{\mathbf{k}}(0)}{\boldsymbol{\psi}_{\mathbf{k}}(0)^*\cdot\boldsymbol{\psi}_{\mathbf{k}}(0)}\right|. \tag{S41}$$

Fig. 1(c) and Fig. 1(d) in the main text show $|C_{-\mathbf{k}}/C_{\mathbf{k}}|$ as a function of $B_{3\mathrm{D}}/\mu$.

B. Discrete Version

Consider a spring mass network that is periodic, and infinite in the x^2 direction and finite in the x^1 direction. For simplicity, we assume the lattice spacing in the x^2 direction to be 1. Such system has two edge bands, and a finite number of bulk bands. We index the bands from low to high frequency by $1, 2, 3, ..., 2N$ (assuming two degrees of freedom per node and N nodes per unit cell) and also index the nodes by $1, 2, 3, ...$ Let $\mathbf{u}^m$ be the displacement vector of the mth node and $\mathbf{u} = (\mathbf{u}^1, ..., \mathbf{u}^N)$ be the displacements of the entire system. We also use $\mathbf{F}^i$ to denote the force on the ith node and $\mathbf{F} = (\mathbf{F}^1, ..., \mathbf{F}^N)$ be the forces on the entire system. We assume an external force $\mathbf{F}^i = \left(F^i_1\cos\omega t, F^i_2\cos(\omega t+\phi)\right)$, where the frequency ω lies in the range of the edge bands, is applied to the ith node at the time $t = 0$. Then, the equation of motion of the system together with initial conditions is

$$m\mathbf{u}'' = -D\mathbf{u} + \mathbf{F},\ \mathbf{u}(0) = \mathbf{0},\ \mathbf{u}'(0) = \mathbf{0}. \tag{S42}$$

To solve this equation, we rewrite $\mathbf{F} = Re\left(\mathbf{F}e^{-i\omega t}\right)$, where the real part of the solution of the resulting complex differential equation is the solution of (S42). Let $\psi_j(\mathbf{k})$ be the normalized Bloch state (where their inner product is the δ function) in the jth band with wave vector $\mathbf{k}$ and corresponding eigenfrequency $\omega_j(\mathbf{k})$. The solution of the equation of motion can be expanded as

$$\mathbf{u}(t) = \int_{-\pi}^{\pi} C_j(\mathbf{k}, t) e^{-i\omega_j(k)t} \psi_j(k). \tag{S43}$$

Plugging (S43) into (S42), we obtain

$$\begin{aligned} \frac{d^2C_j(\mathbf{k},t)}{dt^2} - 2i\omega_j(\mathbf{k})\frac{dC_j(\mathbf{k},t)}{dt} &= \langle\psi_j(\mathbf{k})|\mathbf{F}\rangle e^{-i\left(\omega-\omega_j(\mathbf{k})\right)t} \\ \left.\frac{dC_j(\mathbf{k},t)}{dt}\right|_{t=0} &= C_j(\mathbf{k},0) = 0. \end{aligned} \tag{S44}$$

For a given j and $\mathbf{k}$, if $\omega \neq \omega_j(\mathbf{k})$, $C_j(\mathbf{k}, t)$ is bounded. However, if $\omega = \omega_j(\mathbf{k})$, we have

$$C_j(\mathbf{k}, t) = \frac{i\langle\psi_j(\mathbf{k})|\mathbf{F}\rangle}{\omega} t + q_j(\mathbf{k}, t), \tag{S45}$$

where $q_j(\mathbf{k}, t)$ is bounded. Therefore, at long times, most energy is in the four modes $\psi_1(\mathbf{k}_1)$, $\psi_1(-\mathbf{k}_1)$, $\psi_2(\mathbf{k}_2)$, and $\psi_2(-\mathbf{k}_2)$. Without loss of generality, we can assume the ith node is on the edge and propagating in $+x^2$ direction. The force applied to the ith node needs to follow the trajectory of the motion corresponding to some linear combination of modes $\psi_1(\mathbf{k}_1)$ and $\psi_2(\mathbf{k}_2)$ at node i. Mathematically, that means choosing $\mathbf{F}$ such that it maximizes

$$\frac{|\langle\psi_1(\mathbf{k}_1)|\mathbf{F}\rangle|^2 + |\langle\psi_2(\mathbf{k}_2)|\mathbf{F}\rangle|^2}{|\langle\mathbf{F}|\mathbf{F}\rangle|^2} = \frac{|\langle\psi_1(\mathbf{k}_1)^i|\mathbf{F}^i\rangle|^2 + |\langle\psi_2(\mathbf{k}_2)^i|\mathbf{F}^i\rangle|^2}{|\langle\mathbf{F}^i|\mathbf{F}^i\rangle|^2}. \tag{S46}$$

SIV. ORBITAL AND SPIN ANGULAR MOMENTUM OF PHONONS

In this section, we discuss the general formulation of angular momentum of phonons as vector fields and their orbital and spin components. The main reference of this formulation is Ref. [4]. More sources on this topic include Refs [5, 6].

A. Noether's theorem

Let's start with a brief review of Noether's theorem in field theory, where each continuous symmetry leads to a conserved current.

Consider a generic action S of a set of fields ϕ_a, where $a = 1, \ldots, n$, such that

$$S = \int dx\, \mathcal{L}[\phi_a, \partial_\mu \phi_a], \tag{S47}$$

where we have included in the Lagrangian only the leading-order derivatives of ϕ. To make the notation compact, we use the 4D space-time coordinate $x^\mu \equiv (t, x^1, x^2, x^3)$, where the first component is time and the latter three are coordinates in 3D space. A generic infinitesimal transformation can be written as

$$\begin{aligned} x^\mu \to x'^\mu &= x^\mu + \Delta x^\mu, \\ \phi_a(x) \to \phi'_a(x') &= \phi_a(x) + \Delta\phi_a(x), \end{aligned} \tag{S48}$$

where Δx and $\Delta\phi$ are both caused by the same transformation w, which we can generally write as

$$\begin{aligned} \Delta x^\mu &= \frac{\partial x^\mu}{\partial w_b} w_b, \\ \Delta\phi_a &= \frac{\partial \phi_a}{\partial w_b} w_b. \end{aligned} \tag{S49}$$

Examples of such transformations can be found in Sec IX.

The transformation of the spatial derivatives of the field follows

$$\partial_\mu \phi_a \to \partial'_\mu \phi'_a = \partial_\mu \phi_a - \partial_\nu \phi_a \partial_\mu \Delta x^\nu + \partial_\mu \Delta \phi_a, \tag{S50}$$

where the second term on the right-hand side is from the variation of x, and the last term is from the variation of ϕ, as results from the transformation.

At the same time, the integral over dx' can be written as the integral over $d\mathbf{r}$ with a Jacobian

$$dx \to dx' = dx \det(\partial_\mu x'^\nu) = dx(1 + \partial_\mu \Delta x^\mu). \tag{S51}$$

Putting them all together, we have the transformed action

$$S' = \int dx (1 + \partial_\mu \Delta x^\mu) \mathcal{L}\left[\phi_a(x) + \Delta\phi_a(x), \partial_\mu \phi_a - \partial_\nu \phi_a \partial_\mu \Delta x^\nu + \partial_\mu \Delta \phi_a\right]. \tag{S52}$$

This action can be expanded to leading order in the transformation $\Delta x, \Delta \phi$, so that

$$S' = \int dx \left[\mathcal{L} + \partial_\mu \Delta x^\mu \mathcal{L} + \frac{\partial \mathcal{L}}{\partial \phi_a} \Delta\phi_a(x) - \frac{\partial \mathcal{L}}{\partial(\partial_\mu \phi_a)} \partial_\nu \phi_a \partial_\mu \Delta x^\nu + \frac{\partial \mathcal{L}}{\partial(\partial_\mu \phi_a)} \partial_\mu \Delta\phi_a(x)\right]. \tag{S53}$$

We can also use the Euler-Lagrange equation (the equation of motion of this field theory)

$$\frac{\partial \mathcal{L}}{\partial \phi_a} = \partial_\mu \left(\frac{\partial \mathcal{L}}{\partial(\partial_\mu \phi_a)}\right) \tag{S54}$$

to simplify S' so that

$$S' - S = \int dx \left\{\left[\delta^\mu_\nu \mathcal{L} - \frac{\partial \mathcal{L}}{\partial(\partial_\mu \phi_a)} \partial_\nu \phi_a\right] \partial_\mu \Delta x^\nu + \partial_\mu \left[\frac{\partial \mathcal{L}}{\partial(\partial_\mu \phi_a)} \Delta\phi_a(x)\right]\right\}, \tag{S55}$$

where the first bracket denotes the effect of (external) transformation of coordinate, and the second bracket denotes the effect of (internal) transformation of the field.

Because both of these transformations are caused by the same source $w(x)$, we can use (S49) to rewrite everything in terms of w. In particular, we have terms carrying w and terms carrying $\partial_\mu w$.

Since (S48) can be viewed as simply a coordinate transformation (homogeneous or inhomogeneous), we must have $S' = S$ by definition. On the other hand, when the system has a continuous symmetry such as a homogeneous spatial translation or rotation, the Lagragian (not just the total action) must remain the same before and after the transformation. Thus, the w term must identically vanish everywhere. Therefore, to make $S' = S$, we must also have the integral of the $\partial_\mu w$ terms vanish, which are

$$0 = \int dx \left\{\left[\delta^\mu_\nu \mathcal{L} - \frac{\partial \mathcal{L}}{\partial(\partial_\mu \phi_a)} \partial_\nu \phi_a\right] \frac{\partial \Delta x^\nu}{\partial w_b} + \frac{\partial \mathcal{L}}{\partial(\partial_\mu \phi_a)} \frac{\partial \Delta \phi_a}{\partial w_b}\right\} \partial_\mu w_b. \tag{S56}$$

Because w can be an arbitrary transformation, we move the partial differential via integral by parts and find conserved currents

$$j^\mu_b = \left[-\delta^\mu_\nu \mathcal{L} + \frac{\partial \mathcal{L}}{\partial(\partial_\mu \phi_a)} \partial_\nu \phi_a\right] \frac{\partial \Delta x^\nu}{\partial w_b} - \frac{\partial \mathcal{L}}{\partial(\partial_\mu \phi_a)} \frac{\partial \Delta \phi_a}{\partial w_b}, \tag{S57}$$

which satisfies $\partial_\mu j^\mu_b = 0$.

Now we have proved Noether's theorem, where each continuous symmetry w_b leads to a conserved current j^μ_b satisfying $\partial_\mu j^\mu_b = 0$. Next we discuss its consequence on spatial translations and rotations, the cases of interest in this paper.

B. Translational symmetry

The case of spatial translations can be simply written as translations in the three spatial directions $\mathbf{w} = (w_1, w_2, w_3)$. Thus, $\frac{\partial \Delta x^\nu}{\partial w_b} = 0$ for $\nu = 0$ (time), and δ^ν_b for $\nu = 1, 2, 3$ (space). The $\frac{\partial \phi_a}{\partial w_b}$ terms all vanish as the field itself doesn't transform under spatial translation.

Plugging these into the formula for the conserved current ((S57)), we find:

$$j^0_b = \frac{\partial \mathcal{L}}{\partial(\partial_0 \phi_a)} \partial_b \phi_a, \tag{S58}$$

$$j^i_b = -\delta^i_b \mathcal{L} + \frac{\partial \mathcal{L}}{\partial(\partial_i \phi_a)} \partial_b \phi_a, \tag{S59}$$

where index $i = 1, 2, 3$ runs through spatial indices.

Now, we restore the notation where time and space are separated (instead of the 4D notation), and since all indices are in Euclidean space, we will have them all as sub-indices. We have the resulting continuity equation:

$$\partial_t g_m + \partial_n \tau_{mn} = 0, \tag{S60}$$

which describes *momentum conservation* of this field theory. Here the momentum density g_m and momentum flow τ_{mn} are given by:

$$g_m \equiv -j^0_m, \tag{S61}$$

$$\tau_{mn} \equiv -j^n_m. \tag{S62}$$

This continuity equation (S60) tells us that as a result of spatial translational symmetry, the total momentum of the system

$$P_m = \int d^3x g_m(x) \tag{S63}$$

is a conserved quantity.

C. Rotational symmetry

The case of spatial rotations, which is of central interest to our discussion here, is slightly more complicated. An infinitesimal spatial rotation can be written as $x \to x' = (E + I \cdot w)x$, where E is identity matrix, I denotes the 3 generators of $SO(3)$, and $w = (w_1, w_2, w_3)$ are the angles of rotation in 3D along x, y, z, respectively. This can be written in a more explicit form

$$x'_i = x_i + \varepsilon_{ibj} r_j w_b, \tag{S64}$$

where ε is the Levi-Civita symbol. Thus, we have $\frac{\partial \Delta x_i}{\partial w_b} = \varepsilon_{ibj} x_j$ for the spatial degrees of freedom and $\Delta x^0 = 0$.

How does the field ϕ transform under spatial rotation? This depends on the type of field we consider. Scalar fields won't change. Vector fields, the case of interest here as elasticity is concerned with displacements of components in a solid, should rotate as a vector. This tells us that $\frac{\partial \Delta \phi_a}{\partial w_b} = \varepsilon_{abc} \phi_c$.

Plugging these into the formula of conserved current (S57), we find that for the case of rotation

$$j^0_b = \frac{\partial \mathcal{L}}{\partial(\partial_0 \phi_a)} \partial_\nu \phi_a \varepsilon_{\nu bm} x_m - \frac{\partial \mathcal{L}}{\partial(\partial_0 \phi_a)} \varepsilon_{abm} \phi_m, \tag{S65}$$

$$j^i_b = \left[-\delta^i_\nu \mathcal{L} + \frac{\partial \mathcal{L}}{\partial(\partial_i \phi_a)} \partial_\nu \phi_a \right] \varepsilon_{\nu bm} x_m - \frac{\partial \mathcal{L}}{\partial(\partial_i \phi_a)} \varepsilon_{abm} \phi_m, \tag{S66}$$

where index $i = 1, 2, 3$ runs through spatial indices (and we again wrote them all as sub-indices for simplicity). From the continuity equation, $\partial_\mu j^\mu = 0$, we know that j^0_b offers a new conserved quantity. In particular, this can be written into two parts: the first term, which is from the rotation of the coordinate, and the second term which is from the rotation of the field,

$$-j^0_b = l_b + s_b, \tag{S67}$$

where

$$l_b \equiv \varepsilon_{bmv} x_m g_v, \tag{S68}$$

$$s_b \equiv \varepsilon_{bma} \phi_m \frac{\partial \mathcal{L}}{\partial(\partial_t \phi_a)}. \tag{S69}$$

These two quantities are the orbital and spin angular momenta of the vector field $\boldsymbol{\phi}$. This can also be written as

$$\boldsymbol{l} \equiv x \times \mathbf{g}, \tag{S70}$$

$$\mathbf{s} \equiv \boldsymbol{\phi} \times \frac{\partial \mathcal{L}}{\partial(\partial_t \boldsymbol{\phi})}. \tag{S71}$$

D. Angular momentum of elastic waves

Now we apply this formalism to elasticity, to derive the expressions for the orbital and spin angular momenta [7]. The Lagrangian of a general isotropic solid can be written as

$$\mathcal{L} = \frac{\rho}{2} \partial_t u_i \partial_t u_i - \frac{\lambda}{2} (\partial_i u_i)^2 - \frac{\mu}{2} (\partial_i u_j \partial_i u_j + \partial_i u_j \partial_j u_i)^2, \tag{S72}$$

where ρ is the mass density, μ, λ are the Lame coefficients. Identifying $\mathbf{u}$ as the vector field $\boldsymbol{\phi}$ discussed above, this leads to densities of momentum $\mathbf{g}$, orbital angular momentum $\mathbf{l}$ and spin angular momentum $\mathbf{s}$, where

$$g_i = -\rho \partial_t u_j \partial_i u_j, \tag{S73}$$

$$l_i = \varepsilon_{ijk} x_j g_k, \tag{S74}$$

$$s_i = \rho \varepsilon_{ijk} u_j \partial_t u_k. \tag{S75}$$

These are real physical momentum and angular momentum of the elastic wave. In the vectorial form, the spin angular momentum can also be written as

$$\mathbf{s} = \rho \mathbf{u} \times (\partial_t \mathbf{u}). \tag{S76}$$

From this formula it can be seen that the spin angular momentum of these elastic waves characterize the angular momentum of mass points rotating around their mean positions. In contrast the orbital angular momentum comes from the wave momentum $\mathbf{k}$.

We can apply them to a generic plane-wave field

$$\mathbf{u}(x, t) = \Re \left(\mathbf{A}\, e^{i(\mathbf{k} \cdot x - \omega t)} \right), \tag{S77}$$

where the amplitude A can be a complex vector denoting possible phase difference of different wave components (u_x, u_y, u_z). This leads to

$$\mathbf{g}(x, t) = \rho \omega \left| \Im \left(\mathbf{A} e^{i(\mathbf{k} \cdot x - \omega t)} \right) \right|^2 \mathbf{k} \tag{S78}$$

$$\mathbf{l}(x, t) = \rho \omega \left| \Im \left(\mathbf{A} e^{i(\mathbf{k} \cdot x - \omega t)} \right) \right|^2 x \times \mathbf{k}, \tag{S79}$$

$$\mathbf{s}(x, t) = \frac{1}{2} \rho \omega \Im (\overline{\mathbf{A}} \times \mathbf{A}), \tag{S80}$$

where $\Re$ and $\Im$ denote the real and imaginary parts, respectively, and $\overline{\mathbf{A}}$ is the complex conjugate of $\mathbf{A}$. From this form, it can be seen that $\overline{\mathbf{A}} \times \mathbf{A}$ has to be complex in order for this wave to carry a spin angular momentum.

E. Spin angular momentum of elastic waves in 2D and 3D

We first use these formula to examine the spin of elastic waves in the bulk of isotropic materials. In 3D, the longitudinal waves don't carry spin: their $\mathbf{A}$ is along the wave vector $\mathbf{k}$ so there is only one direction of vibration, leading to $\overline{\mathbf{A}} \times \mathbf{A} = 0$.

Transverse waves in 3D isotropic solids can be generally written as $\mathbf{A} = \{A_1, A_2, 0\}$, taking the direction of k to be the third dimension. Without losing generality we can normalize the amplitude to be unity and factorize out a common phase, so that $\mathbf{A} = \{1, e^{i\theta}, 0\}$. Therefore $\overline{\mathbf{A}} \times \mathbf{A} = \{0, 0, \sin\theta\}$, which is along $\mathbf{k}$ with an amplitude between $[-1, 1]$. Thus $\mathbf{s} = \frac{1}{2}\rho\omega \sin\theta \mathbf{e_k}$ where $\mathbf{e_k}$ is the unit vector along $\mathbf{k}$, from which it can be seen that circularly polarized transverse waves maximize $|\mathbf{s}|$.

In 2D, there are only 2 directions for vibrations, and the wave vector $\mathbf{k}$ is in the same plane. Like in 3D, the 2D medium supports shear and longitudinal waves. In the special case of $B = 0$ in 2D (unlike in 3D), the two waves have the same wavespeeds (and linear dispersion relations). They can be written in a similar form as the 3D case, leading to

$$\mathbf{s} = \frac{1}{2}\rho\omega \sin\theta \mathbf{e_z}, \tag{S81}$$

describing spin angular momentum along the normal direction of the 2D plane. When quantizing this problem, the spin angular momentum of phonons as quanta of these waves take values of $(-\hbar, 0, +\hbar)$. The equality of the longitudinal and transverse wavespeeds in the $B = 0$ case further allows the state of circular polarization to extend into the bulk, as shown in Fig. 1(e). In contrast, for 3D Rayleigh waves, any circular polarization does not extend into the bulk, as illustrated in Fig. 1(f).

SV. BACKSCATTERING-FREE EDGE WAVES AND WINDING NUMBER IN 2D $B/\mu \ll 1$ CONTINUUM

In this section, we discuss in more detail the complex analytic function formulation we used to describe backscattering-free edge waves in 2D $B/\mu \ll 1$ continuum, and the definition of the new winding number.

Consider a 2D homogeneous elastic material with effective bulk modulus $B = 0$ and effective Lamé constants $\lambda = -\mu$ in a bounded simply connected region $\Omega \subset \mathbb{C}$. The equation of motion together with stress free boundary conditions under $B = 0$ is

$$\begin{aligned} \rho\frac{\partial^2 \mathbf{v}}{\partial t^2} &= \mu \nabla^2 \mathbf{v}, \\ n^1\sigma^{11} + n^2\sigma^{12} &= 0, \\ n^1\sigma^{12} + n^2\sigma^{22} &= 0, \end{aligned} \tag{S82}$$

where $\mathbf{n} = (n^1, n^2)$ is the vector normal to the boundary (pointing outward). The in plane displacement vector field $\mathbf{v} = v^1 x_1 + v^2 x_2$ can be identified with a complex scalar field ψ via $\psi = v^1 + iv^2$. We can rewrite the boundary condition as

$$n^1\sigma^{11} + n^2\sigma^{12} + i(n^1\sigma^{12} + n^2\sigma^{22}) = 0. \tag{S83}$$

Under the identification above, the equation of motion together with stress free boundary conditions under $B = 0$ is

$$\begin{aligned} \rho\frac{\partial^2 \psi(z,t)}{\partial t^2} &= 4\mu\frac{\partial^2 \psi(z,t)}{\partial z \partial z^*}, z \in \Omega, \\ \frac{\partial \psi(z,t)}{\partial z^*} &= 0, z \in \partial\Omega, \end{aligned} \tag{S84}$$

where $\frac{\partial}{\partial z} = \frac{1}{2}(\frac{\partial}{\partial x} - i\frac{\partial}{\partial y})$, $\frac{\partial}{\partial z^*} = \frac{1}{2}(\frac{\partial}{\partial x} + i\frac{\partial}{\partial y})$, and $\partial\Omega$ is the boundary of Ω. Note that (S84) admits separation of variables

$$\psi(z,t) = \phi(z)e^{i\omega t} \tag{S85}$$

and

$$\psi(z,t) = \phi(z)e^{-i\omega t}, \tag{S86}$$

where (S85) corresponds to spin-up states (rotate counterclockwise) and (S86) corresponds to spin-down states (rotate clockwise). The profile $\phi(z)$ satisfies

$$\begin{aligned} -\omega^2\rho\phi(z) &= 4\mu\frac{\partial^2 \phi}{\partial z \partial z^*}(z), z \in \Omega, \\ \frac{\partial \phi}{\partial z^*}(z) &= 0, z \in \partial\Omega. \end{aligned} \tag{S87}$$

To solve (S87), we let

$$h(z) = \frac{\partial \phi}{\partial z^*}. \tag{S88}$$

By taking $\frac{\partial}{\partial z^*}$ on both sides of (S87), we see $h(z)$ satisfies Helmholtz equation with Dirichlet boundary conditions [8]

$$\begin{gathered} -\frac{\omega^2 \rho}{4\mu} h(z) = \frac{\partial^2 h}{\partial z \partial z^*}(z), z \in \Omega, \\ h(z) = 0, z \in \partial\Omega. \end{gathered} \tag{S89}$$

Since Ω is a bounded region, it is follows that the eigenvalues $-\frac{\omega^2 \rho}{4\mu}$ are discrete. When $\omega \neq 0$, $h(z)$ has large variation in the bulk and $\phi(z) = -\frac{4\mu}{\omega^2 \rho} \frac{\partial h}{\partial z}$ has large value throughout the bulk, hence it is not an edge mode. When $\omega = 0$, $h(z)$ has unique solution $h(z) \equiv 0$, hence $\frac{\partial \phi}{\partial z^*}(z) = 0$, which is equivalent to $\phi(z)$ being a bounded analytic function. Here, bounded means

$$\exists R > 0, |\phi(z)| < R, \forall z \in \Omega \tag{S90}$$

and analytic means the limit

$$\lim_{u \to 0} \frac{\phi(z+u) - \phi(z)}{u} \tag{S91}$$

exists for all $z \in \Omega$. For $B > 0$, the equation of motion together with the boundary condition becomes

$$\begin{gathered} \frac{\rho}{\mu} \frac{\partial^2 \psi(z,t)}{\partial t^2} = \frac{B}{\mu}\left(2\frac{\partial^2 \psi(z,t)}{\partial z \partial z^*} + \frac{\partial^2 \psi^*(z,t)}{\partial z^2}\right) + 4\frac{\partial^2 \psi(z,t)}{\partial z \partial z^*}, z \in \Omega, \\ \frac{\partial \psi(z,t)}{\partial z^*} = \frac{B}{2\mu} \frac{n^1 - in^2}{n^1 + in^2}\left(\frac{\partial \psi(z,t)}{\partial z} + \frac{\partial \psi^*(z,t)}{\partial z^*}\right), z \in \partial\Omega. \end{gathered} \tag{S92}$$

In the $B \ll \mu$ limit, the B/μ term can be viewed as a perturbation. Let $\psi_{\omega(B/\mu)}(z,t)$ be an eigenmode with frequency $\omega(B/\mu)$, where

$$\lim_{B/\mu \to 0} \omega(B/\mu) = 0. \tag{S93}$$

We have

$$\lim_{B/\mu \to 0} \psi_{\omega(B/\mu)}(z,t) = \phi(z) \tag{S94}$$

for some bounded analytic function $\phi(z)$. If we start from $B/\mu = 0$, then allow some small B/μ, since $\phi(z)e^{i\omega(B/\mu)t}$ and $\phi(z)e^{-i\omega(B/\mu)t}$ serve as zeroth order (in B/μ) solution to (S92), we see

$$\psi_{\omega(B/\mu)}(z,t) \approx \phi(z)e^{i\omega(B/\mu)t} \tag{S95}$$

or we have

$$\psi_{\omega(B/\mu)}(z,t) \approx \phi(z)e^{-i\omega(B/\mu)t}. \tag{S96}$$

Now we prove the following claim: for modes $\phi(z)$ giving rise to waves $\psi_{\omega(B/\mu)}(z,t)$ localized at the boundary, we can find $z_0 \in \Omega$ such that $\phi(z_0) = 0$ and $\phi(z) \neq 0$ when $z \in \partial\Omega$. The claim is a consequence of Rouché's theorem [9], which is: Let Ω be a bounded domain with piecewise smooth boundary $\partial\Omega$. Let $\phi(z)$ and $g(z)$ be two analytic functions that can be analytically extended to an open set containing $\Omega \cup \partial\Omega$. If $|g(z)| < |\phi(z)|, \forall z \in \partial\Omega$, then $\phi(z)$ and $\phi(z) + g(z)$ have the same number of zeros in Ω, counting multiplicities. Mode localized at the boundary has small displacement in the bulk and large displacement at all boundaries, and hence we can find $z_1 \in \Omega$ such that

$$0 < |\phi(z_1)| < |\phi(z)|, \forall z \in \partial\Omega. \tag{S97}$$

We then define $g(z) \equiv -\phi(z_1)$ and consider function

$$f(z) = \phi(z) + g(z). \tag{S98}$$

Since $\phi(z)$ and $g(z)$ satisfy (S97), we can apply Rouché's theorem and see if $f(z)$ and $\phi(z)$ have the same number of zeros (counting multiplicity) in Ω. Since $z = z_1$ is a zero of $f(z)$ in Ω, we know $\phi(z)$ has at least one zero in Ω.

Now we look at the behavior of profile $\phi(z)$ at the boundary. By the argument principle [9],

$$\frac{1}{2\pi i}\int_{\partial\Omega} d\ln(\phi(z)) = \frac{1}{2\pi i}\int_{\partial\Omega} \frac{\phi'(z)}{\phi(z)}dz = N_0 - N_\infty, \tag{S99}$$

where $\frac{1}{2\pi i}\int_{\partial\Omega} d\ln(\phi(z))$ is the number of counterclockwise turns $\phi(z)$ rotates when walking counterclockwise along the boundary (this means Ω is always on the left hand side), N_0 is the number of zeros of $\phi(z)$ (counting multiplicity) inside Ω, and N_∞ is the number of poles of $\phi(z)$ (counting multiplicity) inside Ω. We note that $\phi(z)$ being bounded in Ω implies $N_\infty = 0$ and $\phi(z_0) = 0$ implies $N_0 \geq 1$. Hence, when walking counterclockwise along the boundary, $\phi(z)$ rotates counterclockwise on average. From this, we can see the phase zero point on the boundary in the spin-up solution $\phi(z)e^{i\omega(B/\mu)t}$ moves clockwise and the phase zero point on the boundary in the spin-down solution $\phi(z)e^{-i\omega(B/\mu)t}$ moves counterclockwise, spin and momentum are coupled in $0 < B \ll \mu$ material. Note that this argument doesn't depend on the shape of the material, implying the wave will not be backscattered by defects on the boundary.

SVI. MODE ANALYSIS IN 2D $B/\mu \ll 1$ CONTINUUM MATERIALS

Here, we derive qualitative behaviors of the eigenfrequencies and eigenmode shapes for a homogeneous 2D $B/\mu \ll 1$ continuum material, with Lamé constants $\lambda = -\mu$, within an arbitrary shaped, bounded simply connected region $\Omega \subset \mathbb{C}$.

First, we examine its high frequency behavior. When $B/\mu = 0$, from (S89) we see $\rho\omega^2/\mu$ is the eigenvalue of the laplacian operator $-\nabla^2$ with Dirichlet boundary conditions. Weyl's law states that

$$\lim_{n\to\infty} \frac{N(E)}{E} = \frac{|\Omega|}{2\pi}, \tag{S100}$$

where $N(E)$ is the number of eigenvalues below E and $|\Omega|$ is the area of Ω. Letting ω_n be the nth eigenfrequency, it follows from Weyl's law [8] that

$$\omega_n \approx \sqrt{\frac{2\pi n\mu}{|\Omega|\rho}} \propto \sqrt{n}, \tag{S101}$$

when n is sufficiently large. When $0 < B/\mu \ll 1$, $\omega_n \propto \sqrt{n}$ should still hold as $0 < B/\mu \ll 1$ is only a small perturbation. When the nonzero eigenvalue $\rho\omega^2/\mu$ is present, the eigenfunction $h(z)$ exhibits a large value in the bulk. From (S88), we can deduce that the mode shape $\phi(z)$ also exhibits a large value in the bulk, hence confirming it as a bulk mode. Letting E_1 be the smallest eigenvalue of (S89), in the nonzero but $B/\mu \ll 1$ limit, the bulk mode first appears at frequency $\sqrt{\frac{E_1\mu}{\rho}}$.

Second, we examine frequency behavior below $\sqrt{\frac{E_1\mu}{\rho}}$ for $0 < B/\mu \ll 1$. Letting k_n be the wave vector of the nth mode along the boundary, we then have

$$k_n L = 2\pi N_0(n), \tag{S102}$$

where L is the perimeter of Ω and $N_0(n)$ is the number of turns $\phi_n(z)$ (the nth mode) winds. From

$$\frac{\omega_n}{k_n} = v_{\text{phase}}, \tag{S103}$$

where

$$v_{\text{phase}} = \xi(h)\sqrt{\mu/\rho} \tag{S104}$$

is the phase velocity satisfying (S34) with $h = B/\mu$, as before, we know

$$\omega_n = \frac{2\pi N_0(n) v_{\text{phase}}}{L}. \tag{S105}$$

Since its geometric shape doesn't have any spatial symmetry, ω_n, in general, can only have double degeneracy (corresponding to the degeneracy of spin up and spin down states) and the $(2n-1)^{th}$ edge mode and the $2n^{th}$ edge mode share a common mode shape $\phi_n(z)$. Assuming there is only one $\phi_n(z)$ for each n (we have not been able to establish a rigorous relation for this), we then have $N_0(n) = \frac{n-2}{2}$ for even $n > 3$, $N_0(n) = \frac{n-3}{2}$ for odd $n > 3$ and $N_0(n) \approx \frac{n}{2}$. Therefore, at frequency below $\sqrt{\frac{E_1 \mu}{\rho}}$, we have

$$\omega_n = \frac{\pi n v_{\text{phase}}}{L} \propto n. \tag{S106}$$

The simulation in Fig. 2(a) of the main text uses $L = 1.48$ m, $B_{3D}/\mu = 0.01$. In that case, $v_{\text{phase}} = 21.33$ m/s and

$$\omega_n = \frac{\pi n v_{\text{phase}}}{L} = 45.36n, \tag{S107}$$

which agrees with the simulated fitting slope (43.33) of the green dots in Fig. 3(a). Since we have $N_0(n) \approx \frac{n}{2}$, the mode shape of the $(2n-1)^{th}$ and $2n^{th}$ edge mode $\phi_n(z)$ has n zeros according to (S99). Hence,

$$\phi_n(z) = a_n z^n + ... + a_1 z + a_0 \tag{S108}$$

with some large a_n, because the n zeros lie inside Ω. Since the function z^n is more localized at $\partial\Omega$ than z^m for $m < n$, we know that high frequency edge modes are more localized, which agrees with Fig. 3(b) in the main text.

SVII. LONG WAVELENGTH BEHAVIOR IN MAXWELL LATTICES

In Fig. 4(b) in the main text, we have identified two seemingly overlapping branches, which we denoted as Rayleigh modes. We note that, particularly at long wavelengths (small $\mathbf{k}$), they can also be considered as Lamb modes due to the presence of the two free surfaces (right and left sides of the simulated domain shown in main text Fig. 4(d)). In Fig. S1(a,c), we show the dispersion for two 2D strips of moduli $B_{3D}/\mu > 0$ with periodic boundary conditions on the top and bottom and free boundary conditions on the left and right surfaces (simulated geometries shown in Fig. S1(b,d)). Figures S1(a,b) are of a continuum and Figs. S1(c,d) are of the same Maxwell lattice strip considered in the main text Fig. 4(b-d), but focusing in on low frequencies and wavenumbers. At small $\mathbf{k}$, we can now see that the two Rayleigh modes split into two Lamb modes: one characterized by antisymmetric behavior and parabolic dispersion, and another characterized by symmetric behavior with linear dispersion. At small $\mathbf{k}$, the symmetric mode approaches the longitudinal wavespeed. The higher branches, which we referred to as bulk modes, within the finite context can also be thought of as higher order Lamb modes.

There are a few other noteworthy properties concerning these Lamb waves at small $\mathbf{k}$. First, a significant distinction exists between 2D and 3D scenarios. In 3D, Lamb waves always exhibit quadratic dispersion at small $\mathbf{k}$, regardless of the value of B. However, in 2D, Lamb waves only arise when B is finite. At $B = 0$ in 2D, there is no hybridization between the Rayleigh waves at the two edges, and thus Lamb waves are not observed. Second, the issue of hybridization between modes localized to two independent edges or domain walls is not unique to our study or system. Such hybridization arises in various systems, including quantum Hall and quantum valley Hall edge states. When two 1D edges or domain walls are in close proximity, their hybridization leads to the formation of symmetric and anti-symmetric modes, subsequently resulting in a loss of one-way transport. Often such effects are neglected, parameters and geometries are chosen where this hybridization is weak. In our study, we follow the same approach, ensuring the parameter range studied avoids significant hybridization, namely by avoiding small $\mathbf{k}$ and approaching $B = 0$. However, it is important to note that except for the continuum case idealized to $B = 0$ or infinitely small wavelengths, such hybridization will contribute to reduction of the edge mode backscattering immunity.

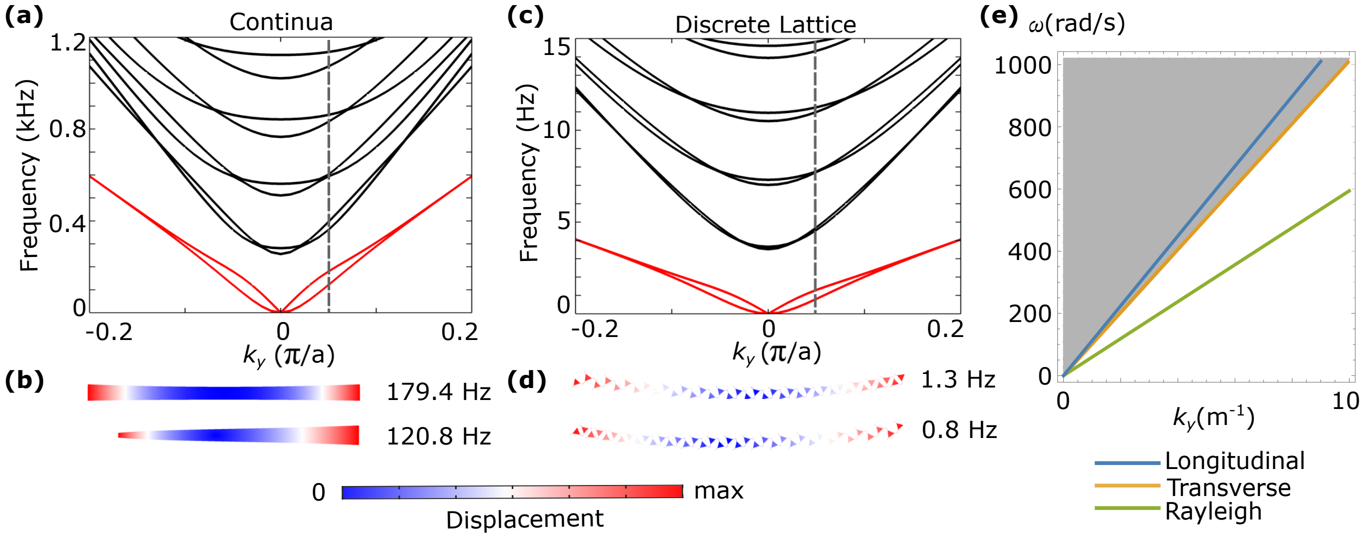

FIG. S1. Dispersion relations of a 2D material strip in the low-k regime for **(a)** a continua strip ($B_{3D}/\mu = 0.1$) and **(c)** a discrete lattice ($B_{3D}/\mu = 0.173$), where the strip is free on left and right boundaries and periodic on the top and bottom. **(b,d)** Mode profiles at $k = 0.05\pi/a$. **(e)** Dispersion of longitudinal, transverse, and Rayleigh waves in 2D continua at $B_{3D}/\mu = 0.1$ (analytic). The shaded area represents bulk modes.

SVIII. BROADBAND EXCITATION OF ONE-WAY EDGE WAVES

Unlike other topologically protected edge modes, the edge modes in this study are not confined to gaps between bulk bands. Instead, for our case of a 2D continuum half space, the edge modes exist for an infinitely broad range of frequencies. In Fig. S2, we demonstrate a one-way edge wave excited by a broadband clockwise Gaussian pulse. The Fourier transform of the excitation reveals a broad frequency range with a bandwidth of 200 Hz, as shown in Fig. S2(a). Using a semi-infinite 2D plane with $B_{3D}/\mu = 0.001$, the Gaussian pulse excitation was applied at a point on the bottom edge (Fig. S2 (b)). Time-domain simulations show that the energy propagates to the right. The energy density as a function of time at points A and B further confirms the one-way nature of the edge mode.

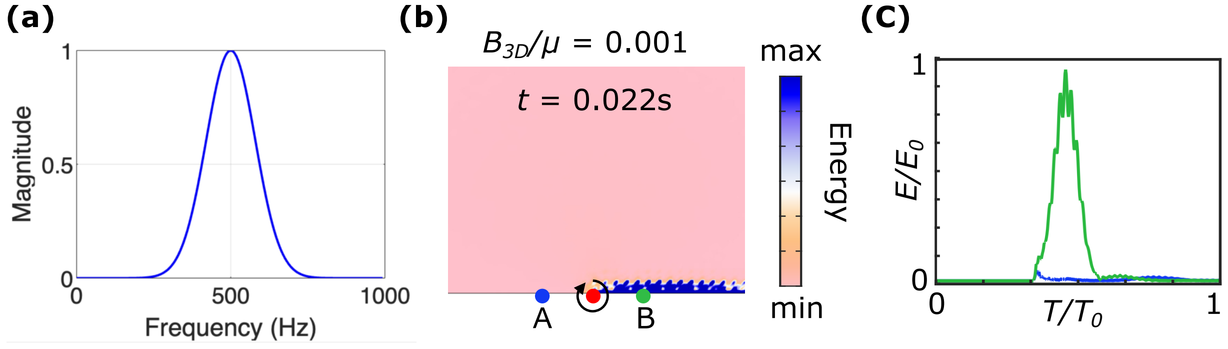

FIG. S2. One-way edge waves with broadband excitation. **(a)** Clockwise Gaussian pulse broadband excitation. **(b)** One-way edge waves in a defect-free semi-infinite geometry ($B_{3D}/\mu = 0.001$). **(c)** Energy density of points A, B (marked in **(b)**) as functions of time.

SIX. BRAGG AND LOCALLY-RESONANT ARRAY SCATTERING OF ONE-WAY EDGE WAVES

In this section, we quantitatively examine the robustness of the backscattering-free one-way transport of the edge waves concerning scatterers resonating with the wave (Bragg scattering) and locally resonant band gaps, where scattering is expected to be the strongest. We show that in the limit of $B/\mu \to 0$, the wave also becomes immune to backscattering in both of these scenarios, because they are protected by the conformal transformation formulasm

discussed in Sec IIB, which is independent of the boundary geometry.

As shown in Fig. S3(a), we start from a semi-infinite plane with a straight edge, and consider a Rayleigh wave with wavelength of 4 cm. We then examine this wave against Bragg scattering, as is demonstrated in Fig. S3, employing a sinusoidal surface roughness with a periodicity of 2 cm, precisely matching half the wavelength. Using a unit cell with periodic boundary conditions as shown in Fig. S3(b), we demonstrate that this results in the appearance of Bragg gaps for the surface wave branch, as can be seen in Fig. S3(c). As B_{3D}/μ decreases and approaches zero, the gap becomes infinitely small. We note that the example of Fig. S3(b,c) is used simply to demonstrate the decreasing bandgap width, as the dispersion is slightly modified due to the finite depth, compared to the time domain simulations we will show as follows. In Fig. S3(d), we show time domain simulations where we inject the same surface wave as Fig. S3(a), but with frequency chosen to provide a wavelength of 4 cm for all B_{3D}/μ. The three non-excited boundaries are set to be absorbing. The simulations are conducted for $T \times 20$ s, where T is the period of the excitation frequency. The excitation is applied as a ramped circularly polarized signal. We see that the excitation in the presence of the periodic structure's band gap creates the formation of a laterally localized standing wave at the surface. It can be seen as B_{3D}/μ approaches zero, the scattering due to the periodic surface roughness is drastically reduced. We suggest there are thus three simultaneous effects here. First, the gap's decrease in width results in weaker attenuation in the gap and a longer decay length. Second, the frequency filtering width is reduced, which is particularly pertinent in the case of any transient broadband excitation. Third, the backscattering immunity strength grows, as it more closely approaches circular polarization.

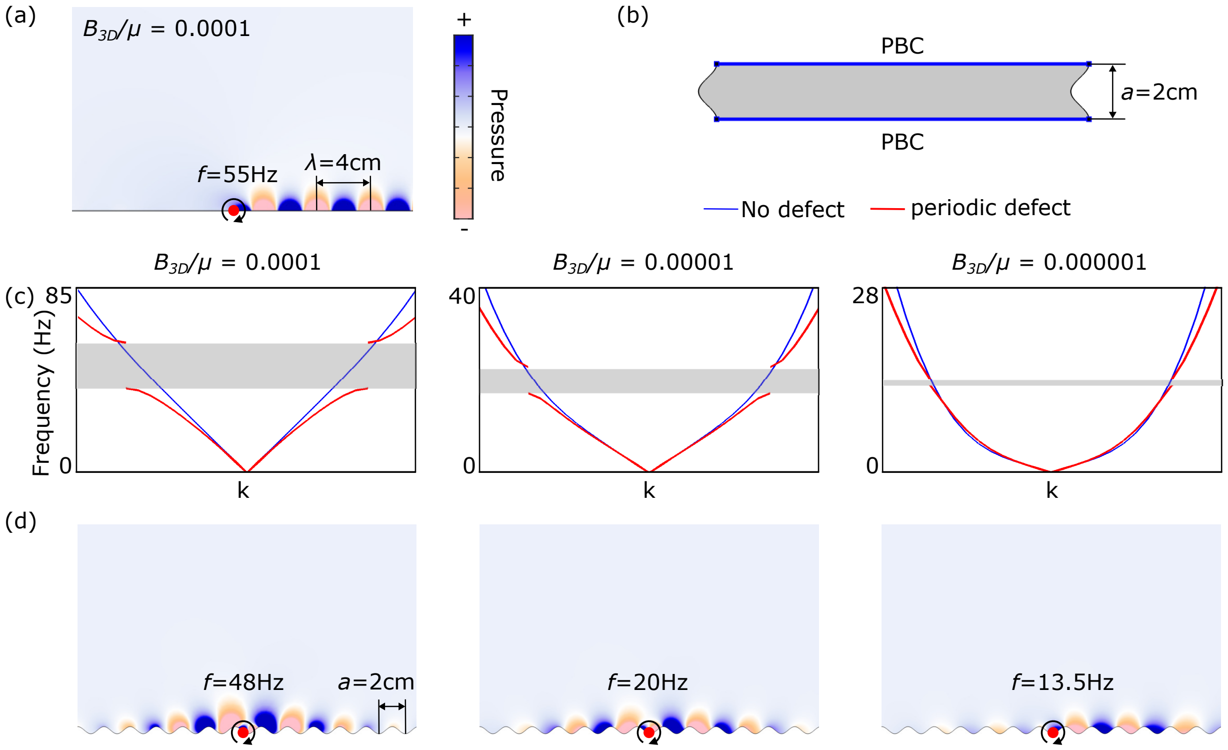

FIG. S3. One-way edge waves encountering structures inducing Bragg (periodic) band gaps. **(a)** One-way edge waves at a frequency of 55.67 Hz in a defect-free geometry ($B_{3D}/\mu = 0.0001$), with a wavelength of 4 cm. **(b)** Periodic roughness geometry used to calculate the dispersion in (c) with periodic boundaries marked in blue. **(c)** The surface wave band structure for $B_{3D}/\mu = 0.000001, 0.00001, 0.0001$. The bands corresponding to the geometry in **(b)** are highlighted in red, while those associated with defect-free geometries are marked in blue. **(d)** Backscattering-free propagation of one-way edge waves in geometries with sinusoidal periodic roughness, where the periodicity (2 cm) equals half the wavelength.

In Fig. S4, we present a second scenario, this time with resonant pillars at the boundary. The depth of the resonant pillar set to 2 cm, resulting in the opening of locally-resonant band gaps. In the dispersion of Fig. S4(a), a similar unit cell is used as in Fig. S3(b). Here, this allows us to identify the frequency of the locally resonant gap with varied B_{3D}/μ. As before, we can see that the gap width narrows as B_{3D}/μ approaches zero. We then use a time domain simulation, as seen in Fig. S4(b), in a similar manner to Fig. S3, wherein we excite at the mid-gap frequency shown in Fig. S4(a). Again it can be seen that as B_{3D}/μ approaches zero, the scattering due to the locally-resonant band gap

is drastically reduced. Finally, we also include additional time domain simulation results in Fig. S4(c) wherein it can be observed that even in the absence of defects on the left side of the excitation, the wave propagates to the right side without backscattering.

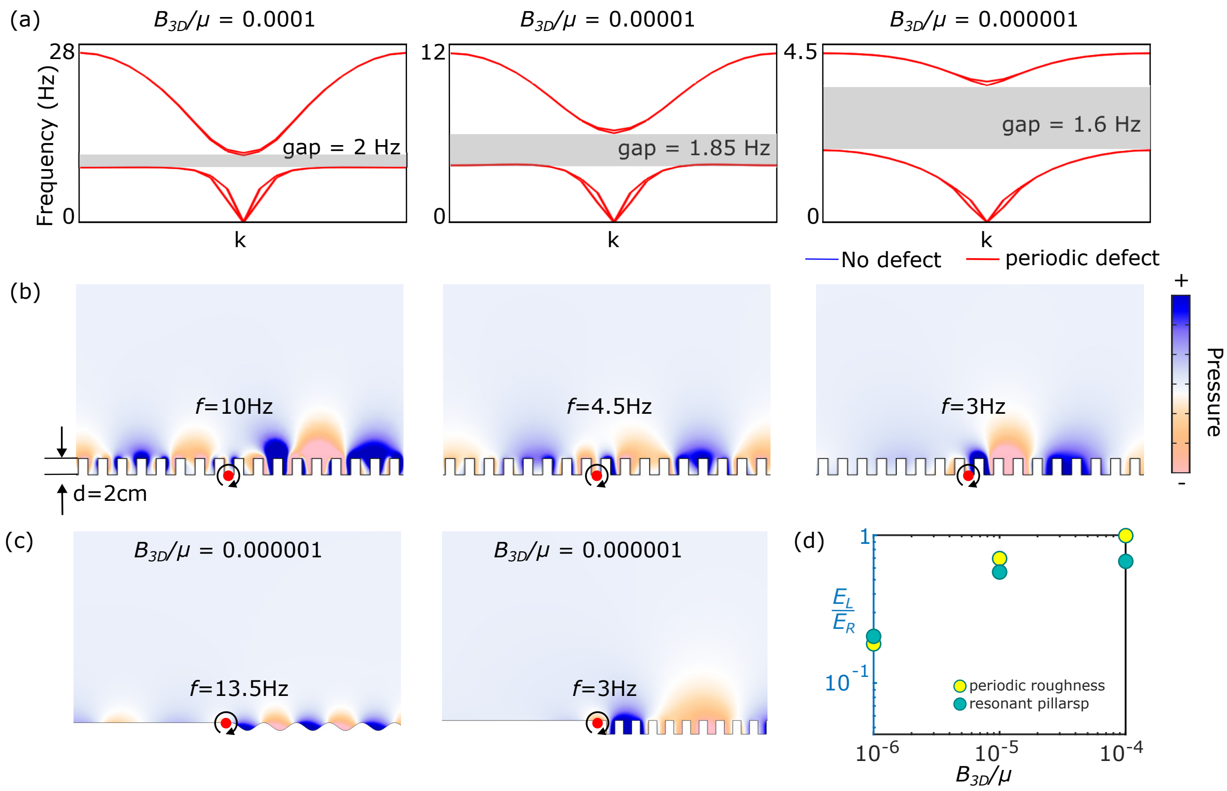

FIG. S4. One-way edge waves encountering structures inducing locally-resonant band gaps. **(a)** The surface wave band structure of elastic continua with resonant pillars roughness for $B_{3D}/\mu = 0.000001, 0.00001, 0.0001$. **(b)** Backscattering-free propagation of one-way edge waves in geometries with resonant pillars, where the excitation frequency falling within the locally-resonant band gap. **(c)** Additional cases with only periodic roughness(left) and resonant pillars (right) on the right side of the excitation.**(d)** The energy ratio between left (L) and right (R) propagating modes (cumulative over time) at different B_{3D}/μ demonstrates backscattering resistance in the limit of $B_{3D}/\mu \to 0$

The results demonstrate that, as long as B_{3D}/μ approaches 0 sufficiently (noting that these band-gap structures require smaller B_{3D}/μ than other types of roughness), the edge waves in the 2D continuous system still exhibit backscattering-free behavior.

SX. ADDITIONAL EXPERIMENTAL DETAILS

A. Mortise and tenon assembly

An example of CNC machined polycarbonate parts for lattice assembly via press fit is shown in Fig.S5.

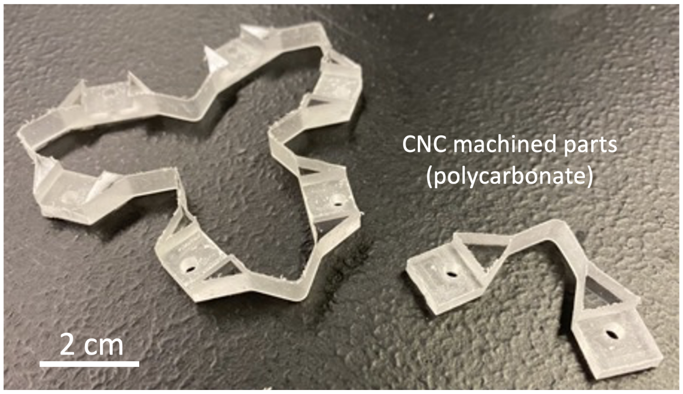

FIG. S5. Example of CNC machined sublattice elements with mortise and tenon structures to form solid triangles.

B. Energy ratio calculation

The energy ratio calculated in experiments between the edge waves propagating in opposite directions is calculated by comparing the sums of the squared velocities of all edge triangles' centroids on the left and right sides of the actuator (shown in Fig. S6) over the time period of 0.01 – 0.15 s, before the two edge waves encounter each other. It should be noted that this ratio does not correspond to the ratio $|C_{-\mathbf{k}}/C_{\mathbf{k}}|$, which predicts the relative amplitude of the opposite propagating modes in the $t \to \infty$.

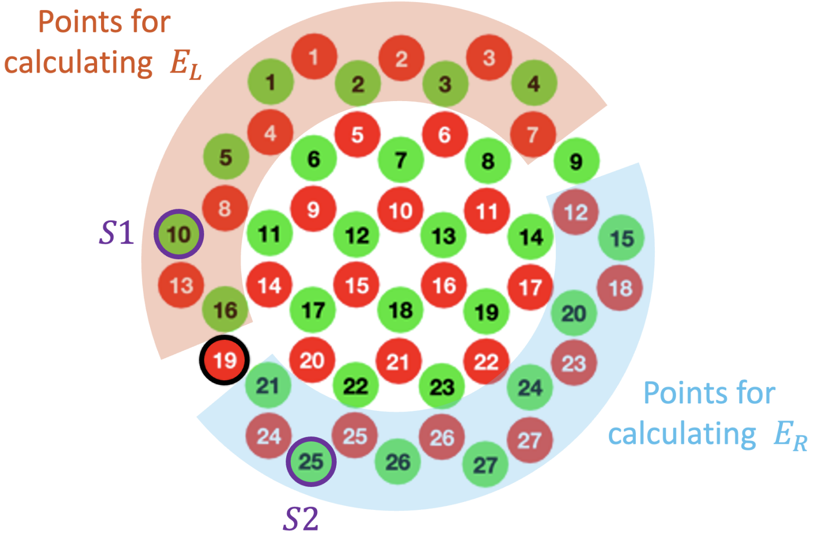

FIG. S6. Triangles on the edge to the left and right of the excitation point (black circle) used for calculating energy ratio E_L/E_R shown in Fig. 4(e-j) and plotting average kinetic energy $\langle v^2 \rangle$ in Fig. S8. Numbers in the red and green solid circles are indices used for data processing in MATLAB. $S1$ and $S2$ mark the triangles on the edges equidistant from the actuated point used for tracking the trajectory phases shown in Fig. S8.

C. Poisson's ratio measurement

The Poisson's ratio of our manufactured Maxwell lattice was measured based on manual compression of the lattice and image tracking of axial compression (perpendicular to the edge) and transverse (lateral) expansion. The fitted slope gives $\nu \simeq -0.488$ (Fig. S7), corresponding to $B/\mu = 0.344$ ($B_{3D}/\mu = 0.173$).

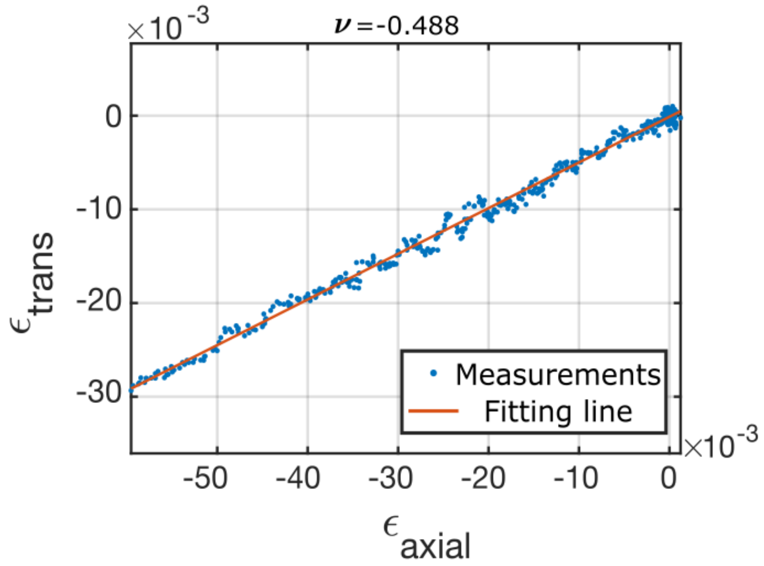

FIG. S7. Measured Poisson's ratio of the auxetic Maxwell lattice. $\epsilon_{\rm axial}$ and $\epsilon_{\rm trans}$ represent the strains along and perpendicular to the compression, respectively.

D. Additional experiment and simulation comparison

In Fig. S8, we present the raw data for the simulations and measurements of spin-momentum locking and one-way edge waves in Maxwell lattices, which were reported in the main text Fig. 4 (e-j). As explained in the main text, it was observed that the opposite propagating waves exhibit opposite spins. To further analyze this phenomenon, the velocity phases ($\arg(v)$) of S1 and S2 were examined. It was consistently found that S1 rotates in a clockwise direction, while S2 rotates counterclockwise before the two edge waves encounter each other. This observation confirms the presence of spin-momentum locking in the edge waves. To quantify the energy distribution between the left- (L) and right- (R) propagating edge modes, the energy ratio $E_{\rm L}/E_{\rm R}$ was calculated. This ratio was obtained by integrating the average squared velocity (over the time range $0.01 - 0.15$ s) of the triangles located on the edge to the left and right of the excitation point, as depicted in Fig. S6. Considering that the group velocity at 5.5 Hz is approximately 0.03 m/s and the distance from the actuation source to the intersection of the two waves is around 0.45 m, the two edge waves encounter each other after 0.15 s.

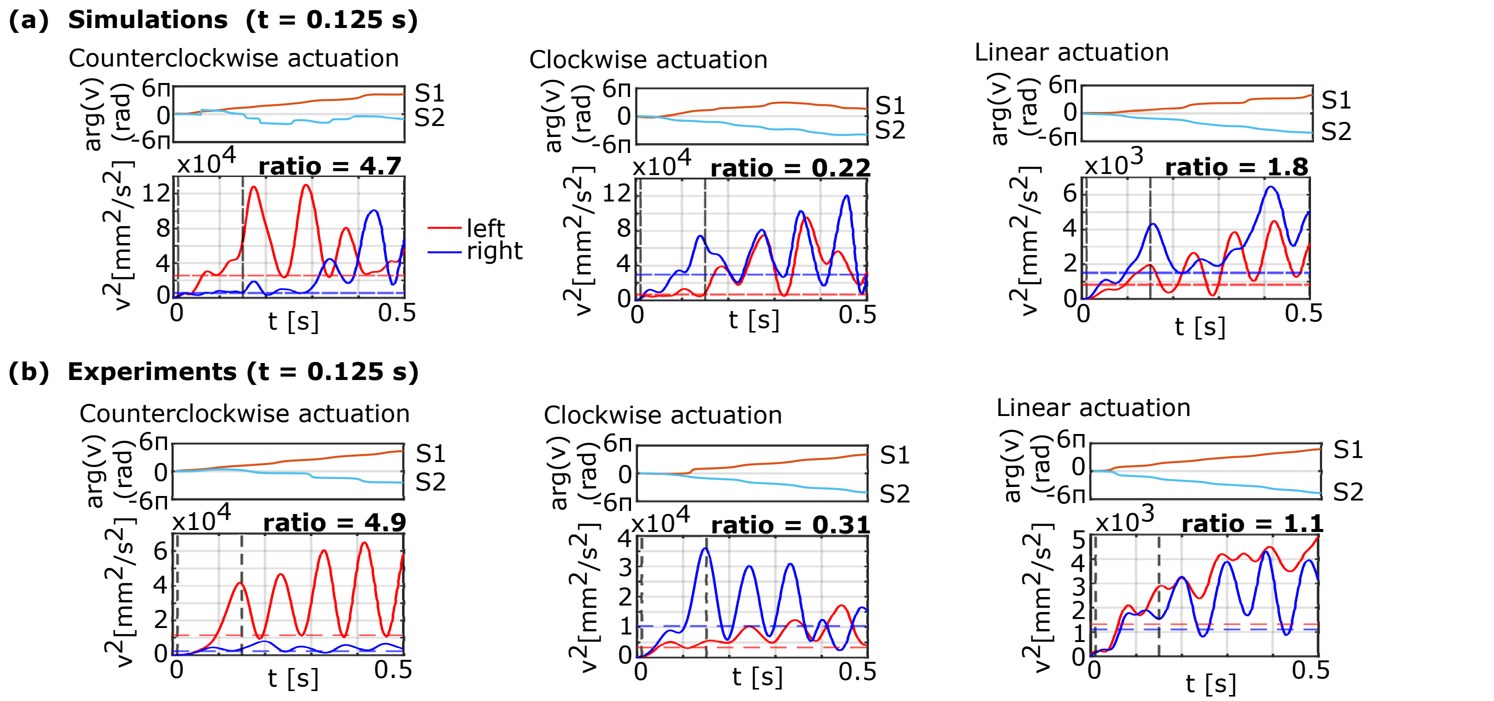

FIG. S8. Experiments and simulation raw data for one-way edge waves in Maxwell lattices. **(a)** Time domain simulations and **(b)** experimental measurements of one-way edge waves in auxetic Maxwell lattices. In each panel, the top figures display the velocity vector phases (arg(v), which are shifted to start from 0) of the centroids of triangles S1 and S2 (as indicated in Fig.S6) in the time domain under different excitation types. The bottom figures illustrate the average kinetic energy (v^2) in the same time range, calculated based on the triangles on the edge, as shown in Fig. S6. The term 'ratio' represents the energy ratio E_L/E_R.

SXI. ADDITIONAL MAXWELL LATTICE SCATTERING SIMULATIONS

Additional FEM simulations are performed, where we deliberately introduce disorder in our simulated Maxwell lattice by removing or adding unit cells at the boundary or creating cavities within the bulk. As depicted in Fig. S9, the results demonstrate that this does not significantly disrupt the one-way edge transport along the edge.

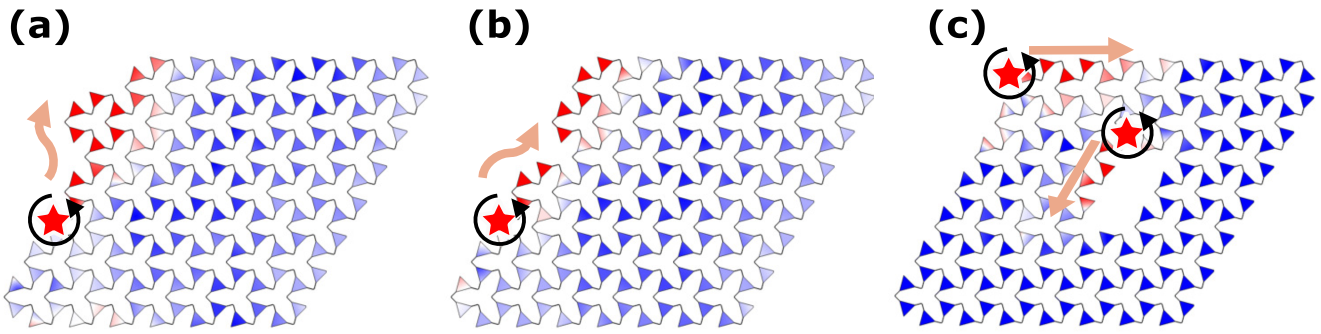

FIG. S9. **(a-c)** Robustness of edge wave transport in the auxetic Maxwell lattice by simulating various types of defects, where three triangles were added to the left edge (**(a)**), three triangles were removed from the left edge (**(b)**), and four triangles were removed from the bulk (**(c)**). The excitation position is denoted by a red asterisk, and actuation is carried out in a counterclockwise direction.

[1] L. Landau, E. Lifshitz, A. Kosevich, and L. Pitaevskii, *Theory of Elasticity: Volume 7*, Course of theoretical physics (Elsevier Science, 1986).
[2] K. F. Graff, *Wave motion in elastic solids* (Courier Corporation, 2012).
[3] J. Oliver, F. Press, and M. Ewing, Two-dimensional model seismology, Geophysics **19**, 202 (1954).
[4] P. Francesco, P. Mathieu, and D. Sénéchal, *Conformal field theory* (Springer Science & Business Media, 2012).
[5] D. Tong, Lectures on quantum field theory, Part III Cambridge University Mathematics Tripos, Michaelmas (2006).
[6] A. Beekman, L. Rademaker, and J. van Wezel, An introduction to spontaneous symmetry breaking, SciPost Physics Lecture Notes , 011 (2019).
[7] J. Ren, From elastic spin to phonon spin: symmetry and fundamental relations, Chinese Physics Letters **39**, 126301 (2022).
[8] W. Strauss, *Partial Differential Equations: An Introduction* (Wiley, 2007).
[9] T. Gamelin, *Complex Analysis*, Undergraduate Texts in Mathematics (Springer New York, 2003).
